# Evolving therapeutic paradigms in ocular graft-versus-host disease

**DOI:** 10.1038/s41433-024-03311-x

**Published:** 2024-08-24

**Authors:** Mouayad Masalkhi, Noura Wahoud, Bridget Moran, Ezzat Elhassadi

**Affiliations:** 1 School of Medicine, University College Dublin, Belfield, Dublin, Ireland; 2 Faculty of Medicine, American University of Beirut, Beirut, Lebanon; 3 Mater Miscordiae University Hospital, Dublin, Ireland; 4 Department of Hematology, University Hospital Waterford, Waterford, Ireland

**Keywords:** Biologics, Biomarkers

## Introduction

Graft-versus-host disease (GVHD) is a complex immune-mediated condition that arises when donor T-cells recognize recipient antigens as foreign, which culminates to a pathological immune response [1]. The pathophysiology of aGVHD involves a cascade of events starting with tissue damage and the subsequent cytokine-mediated activation of antigen-presenting cells (APCs) [2]. Chronic GVHD (cGVHD) may involve additional mechanisms such as thymic injury, B cell autoantibody synthesis, and the formation of profibrotic lesions [2].

Ocular GVHD (oGVHD) is a significant complication of GVHD, presenting with a range of ocular symptoms including dry eyes, irritation, redness, and more severe manifestations such as corneal ulcers and perforation. Adnexal features of oGVHD like poliosis, lagophthalmos, vitiligo, and entropion are also reported [3]. Ocular hypertension is a common yet typically transient complication, often resolving with the reduction of corticosteroid doses [3]. However, serious complications such as persistent epithelial defects and irreversible vision loss can occur, which highlights the importance of effective management strategies. Therefore, early detection and interdisciplinary approaches are crucial for improving clinical outcomes and quality of life for patients with oGVHD. In this paper, we aim to discuss the pathophysiology and clinical manifestations of oGVHD with an emphasis on current and future management strategies due to their increasing importance.

## Pathophysiology and clinical manifestations of oGVHD

The driving mechanism behind complications seen in oGVHD is a T-cell response to specific proteins such as human leukocyte antigens (HLA) on the surface of host cells [3]. Acute GVHD (aGVHD), defined as GVHD is believed to occur as a three-step process [4]. Tissue damage leads to cytokine-mediated activation of APCs [5]. This is followed by donor T-cell “activation, proliferation, differentiation and migration”, which lead to further T-cell medication tissue damage [5]. Another hypothesis suggests the interplay of thymic injury, B cell autoantibody synthesis and profibrotic lesion formation—that serves to perpetuate the inflammatory insult that occurs in cGVHD [6].

Clinically, GVHD can manifest with ocular features such as dry eyes, irritation, and redness due to underlying inflammation of mucosal surfaces [4]. Adnexal features of GVHD include poliosis, lagophthalmos, vitiligo, and entropion [4]. Corneal GVHD may present with filamentary keratitis, superficial punctate keratopathy, corneal ulcers or in severe cases, corneal perforation [7]. Ocular hypertension is a common complication of oGVHD; however, it is often transient and resolves with decreasing the corticosteroids dose received [8]. Though rare, serious complications of oGVHD include corneal perforation, conjunctival scarring, persistent epithelial defects and irreversible vision loss [9].

## Novel advances and therapies

### Lacrimal gland

oGVHD manifests as interstitial inflammation and fibrosis in the lacrimal gland and ultimately results in dry eye disease if left untreated [10]. Molecular targeting HSP47, which plays a critical role in LG fibrosis, was found to result in decreased restored tear secretion in a mouse model with oGVHD [11]. This finding has opened the door to test drugs targeting HSP47, with valsartan showing promising results [12]. Tranilast, used to treat scleroderma-associated fibrosis, showed effectiveness in managing LG fibrosis in oGVHD in a clinical trial [13]. Recent studies have also shown that targeting senescence-associated secretory phenotype-associated cytokines, such as IL-6 and CXCL9, may help treat lacrimal gland dysfunction in oGVHD mouse models [14].

### Cornea

Corneal impairment in oGVHD can affect all the corneal layers, and can result changes ranging from endothelial cell damage and stromal oedema to corneal epithelial atrophy and necrosis in severe cases [10]. Recently, the reduction in corneal endothelial cell density in oGVHD has been speculated to involve the substance P/neurokinin-1 receptor (NK1R) pathway [15]. targeting this pathway with the NK1R antagonist Fosaprepitant was studied in a mouse model and demonstrated a significant reduction in corneal epithelial damage and immune cell infiltration [15]. oGVHD can also impact the corneal neurosensory layer, leading to neurotrophic ulcers through abnormal activation of the complement C3/CD4 + T-cell axis [16]. Notably, targeting C3 with a cobra venom-derived drug provided corneal protection [16]. Umbilical cord serum and plasma rich in growth factors have been utilized to support corneal epithelial healing, achieving high rates of ulcer resolution [17, 18]. Topical insulin has recently been shown to be superior to autologous serum in the treatment of persistent epithelial defects [19]. Scleral and bandage soft contact lenses have also demonstrated to be effective in treating refractory ocular surface conditions in oGVHD [20, 21]. Amniotic membrane transplantation has recently emerged as a highly valuable procedure for severe and refractory cases of oGVHD [22].

A vascular adhesion protein-1 (VAP-1) inhibitor and subconjunctival injection of AAV-HLA-G (a therapy of adeno-associated virus (AAV) gene transfer of HLA-G) have mitigated oGVHD-associated inflammation and fibrosis [23, 24]. Inhibition of JAK 1/2 via Ruxolitinib has shown promise in the treatment of both steroid-resistant and steroid-dependent oGVHD [25, 26]. A clinical trial revealed that eyes with refractory oGVHD-associated DED that were treated with exosomes from mesenchymal stromal cells (MSC-exo) had substantial relief [27]. Diquafosol ophthalmic solution, an eye drop for mucin production and water secretion, has been shown to be both effective and safe for the treatment of oGVHD-DED [28]. Lifitegrast, a lymphocyte function-associated antigen-1 antagonist, is another novel therapy that has been demonstrated to be safe and effective in treating oGVHD manifesting as keratoconjunctivitis sicca [29–33].

## Conclusion

Overall, oGVHD is a one of the most common and potentially debilitating, complications following ASCT, where it can significantly impact quality of life. Symptoms for oGVHD range from redness and itching to corneal perforation and irreversible vision loss. Early detection and an interdisciplinary approach are crucial for the effective management and improved clinical outcomes of oGVHD. Furthermore, the development of novel therapies and algorithms to aid in earlier detection of the disease is rapidly advancing, particularly with the growing role of machine learning and artificial intelligence in clinical and diagnostic ophthalmology.
